# Multi-echo versus single-echo EPI sequences for task-fMRI: A comparative study

**DOI:** 10.1162/IMAG.a.94

**Published:** 2025-07-28

**Authors:** Alice Giubergia, Giulio Ferrazzi, Marco Castellaro, Sara Mascheretti, Valentina Lampis, Florian Montano, Alessandra Bertoldo, Denis Peruzzo

**Affiliations:** Scientific Institute IRCCS Eugenio Medea, Neuroimaging Unit, Bosisio Parini, Italy; University of Padova, Department of Information Engineering, Padova, Italy; Philips Healthcare, Milan, Italy; University of Pavia, Pavia, Italy; Scientific Institute IRCCS Eugenio Medea, Radiology & Neuroradiology Unit, Bosisio Parini, Italy

**Keywords:** fMRI, task-fMRI, multi-echo fMRI, reading task

## Abstract

Functional magnetic resonance imaging (fMRI) studies in cognitive and clinical neuroscience rely on blood oxygenation level-dependent (BOLD) contrast, measured with single-shot gradient-echo-planar imaging. However, conventional schemes encompass the acquisition of single-echo fMRI, which samples a single echo at a single-echo time (TE), facing limitations in disentangling neural signals from artifacts. Multi-echo (ME) fMRI captures images at multiple echo times within a single repetition time (TR) period and enables the separation of BOLD and non-BOLD signal components. Previous studies have highlighted the benefits of ME-fMRI but often relied on comparisons with suboptimal single-echo data derived from ME acquisitions, limiting the validity of these evaluations. This study performs a more rigorous comparison between three datasets: the data acquired with an optimized single-echo (OSE) fMRI sequence at the highest possible temporal resolution, those acquired with an ME-fMRI sequence, and, as previously reported in the literature, the echo-2 time-series extracted from the ME-fMRI data itself. ME-fMRI *vs.* echo-2 comparison confirmed previous findings, which advantage the ME approach. However, the acquisition of multi-echo fMRI did not clearly outperform an optimized single-echo scheme. While OSE-fMRI exhibits benefits in terms of higher statistical power, ME-fMRI demonstrates superior performance at the single-subject level in terms of reliability (p < 0.05). Additional investigation and optimization could clarify the conditions under which one sequence may be preferred over the other.

## 1 Introduction

The vast majority of functional magnetic resonance imaging (fMRI) studies in cognitive and clinical neuroscience use blood oxygenation level-dependent (BOLD) contrast as a proxy for brain activity, whether experimentally induced or spontaneous. BOLD contrast is most commonly measured using single-shot gradient-echo-planar imaging (single-shot EPI; Mansfield, 1977), an imaging technique sensitive to variations in the relaxation time T2* (=1/R2*) caused by changes in deoxyhemoglobin levels in the brain (Bandettini et al., 1992; Kwong et al., 1992; Ogawa et al., 1992). In conventional fMRI, the signal is sampled at a (single)-echo time (TE). However, single-echo (SE) fMRI faces a fundamental challenge: the indeterminacy of signal sources. The signals recorded during fMRI are influenced by a complex mixture of neural and non-neural factors, making it challenging to discriminate “true” neural activity from other sources, such as motion and/or physiological noise, which in turn influence effect size and statistical power in the analysis of both task and resting-state fMRI data (Kundu et al., 2017). Standard fMRI approaches employ data from one echo time and, therefore, lack a general ground truth to explain biophysical mechanisms with acquired signals and to disentangle artifacts, because there is not enough information to separate these effects based on their different T2* decay characteristics (Kundu et al., 2017).

In contrast, multi-echo (ME) fMRI has been proposed to improve the fidelity of fMRI signals through a physically driven determination of the origins of fMRI (Kundu et al., 2017; Lombardo et al., 2016). In ME-fMRI, images are acquired at different echo times after a single radio-frequency (RF) excitation (i.e., within the same TR), allowing T2* quantitative values to be estimated from a mono-exponential decay model. As BOLD signals arise by changes in T2* over time (whereas TE-independence characterizes non-BOLD signals), T2* signal decay can be modeled to distinguish BOLD from artifacts components and infer brain activity (Kundu et al., 2017).

Methodological studies have reported the beneﬁts of ME-fMRI (Gilmore et al., 2022; Heunis et al., 2021) when compared with SE; however, those analyses were performed using, as SE, the time-series extracted from ME data itself, which is suboptimal as this leads to artificial SE data but acquired with a much lower temporal resolution. Thus, a comparison with optimized single-echo (OSE) data derived from an independent acquisition with a shortest possible time of repetition (TR) must be carried out to get a fair evaluation of the possible advantages of ME over SE acquisitions.

In this study, a thorough comparison between ME-fMRI *vs* OSE-fMRI is investigated. Our rationale aims to determine whether ME acquisitions might actually lead to improved detectability of brain activity over OSE which in turn translates into a significant amelioration in data quality and interpretability (Gilmore et al., 2022; Gonzalez-Castillo et al., 2016; Kundu et al., 2012, 2017).

## 2 Materials and Methods

This study was approved by the Scientific Review Board and the Ethical Committee of the Scientific Institute, IRCCS Eugenio Medea.

### 2.1 Participants

Twelve healthy Italian native speakers (age = 28 ± 5; 4 males) provided written informed consent to participate in the present study. All anatomical scans were inspected by a radiologist and no incidental findings were reported.

### 2.2 Experimental and task design

The experimental protocol included one reading task administered multiple times during the acquisition of the OSE-fMRI and ME-fMRI sequences.

The task was designed to study the involvement of the visual dorsal pathway when reading visually and spatially manipulated words. The task-based fMRI design consisted of 192 trials per run, during which participants were asked to read in their minds the words on the screen. Each trial began with a 200 ms fixation cross, followed by a stimulus lasting 1.8 s. The experiment was split into two runs and implemented using Presentation Software® (Neurobehavioral System Inc., Berkeley, CA, USA) version 24.0 and delivered through a VisuaStim digital device for fMRI (Resonance Technology Inc., Northridge, CA, USA).

**Fig. 1.**
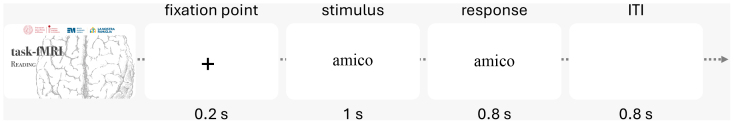
Trial timeline. In each trial, the stimulus is introduced by a fixation cross. The response window begins at the stimulus presentation and lasts 0.8s more. Finally, a fixed 0.8s ITI was included.

Participants were instructed to press a button whenever a catch trial was presented (any word representing animals) to ensure they were engaged in performing the task. The response window lasted 1.8 s (including the stimulus appearance) and the post-response inter-trial interval (ITI) was set to 0.8 s and lasted until the onset of the next stimulus, which was synchronized with the scanner trigger (TR) (Fig. 1). Stimuli consisted of 24 high-frequency 3-syllable nouns selected from the Italian lexicon on the basis of (a) word structure (flat syllables or not; presence of consonant clusters), (b) orthographic words, (c) length, and (d) category (abstract/concrete words) (see Supplementary Table S1 for the complete list of selected words). Black stimuli in Times New Roman font size 130pt were presented on a white background and with different visual and spatial transformations (see Table 1). Each combination (word, spatial, and visual transformations) was presented once.

**Table 1.** Stimulus design.

	Transformation of the baseline			
	Spatial transformation			Visual transformation
Levels	Rotation [°]	Mirror	Spacing	Contrast [%]
	±15	Left-right (LR)	2	50
	±30	Upside-down (UD)	4	25
	±45	LRUD	6	15

List of the spatial transformations and degradation levels implemented in the reading task. Each combination of spatial transformation and degradation level was presented once for each word.

### 2.3 MRI acquisition protocol

MRI data were acquired on a 3T Philips Achieva d-Stream scanner (Best, The Netherlands) with a 32-channel receiver head coil. A T1-weighted (T1-w) anatomical image was acquired using a 3D Magnetization Prepared Rapid Gradient Echo (MP-RAGE) sequence as a subject morphological reference (Field Of View (FOV) = 240 x 240 x 181 mm^3^, voxel size = 1 x 1 x 1 mm^3^, TR = shortest (~6.9 ms), TE = shortest (~3.2 ms), Inversion Time (TI) = 1060 ms, Flip Angle (FA) = 8°) together with a FLAIR 3D sequence (FOV = 247 x 247 x 183 mm^3^, voxel size = 1.12 x 1.12 x 1.12 mm^3^, TR = 8s, TE = 360 ms, FA = 40°, TI = 2400 ms). The OSE-fMRI data were acquired with a T2*-weighted single-shot EPI sequence (FOV = 240 x 240 mm^2^, voxel size = 2.5 x 2.5 mm^2^, slice thickness = 2.5 mm, number of slices = 56, multiband factor = 4, SENSE = 1.9, TR = 1100 ms, TE = 23 ms, FA = 50°). The ME-fMRI data were acquired with a single-shot EPI ME sequence (FOV = 240 x 240 mm^2^, voxel size = 3 x 3 mm^2^, slice thickness = 3 mm, slice gap = 0.3 mm, number of slices = 40, TR = 1650 ms, TEs = 14-40-66 ms, multiband factor = 4, SENSE = 2, FA = 74°). OSE-fMRI and ME-fMRI sequences were acquired twice to include both runs of the reading task: ME-run1, OSE-run2, OSE-run1, and ME-run2, presented in pseudorandom order. The number of trials presented during the experimental task was the same in every acquisition, although the time allocated to each trial could slightly vary depending on the participant’s response speed to catch trials, with a maximum limit of 800 ms (Fig. 1). In addition, two spin-echo sequences with opposite phase encoding directions (anterior-posterior/posterior-anterior) were acquired for B0 distortions estimation purposes.

### 2.4 MRI data preprocessing

**Fig. 2.**
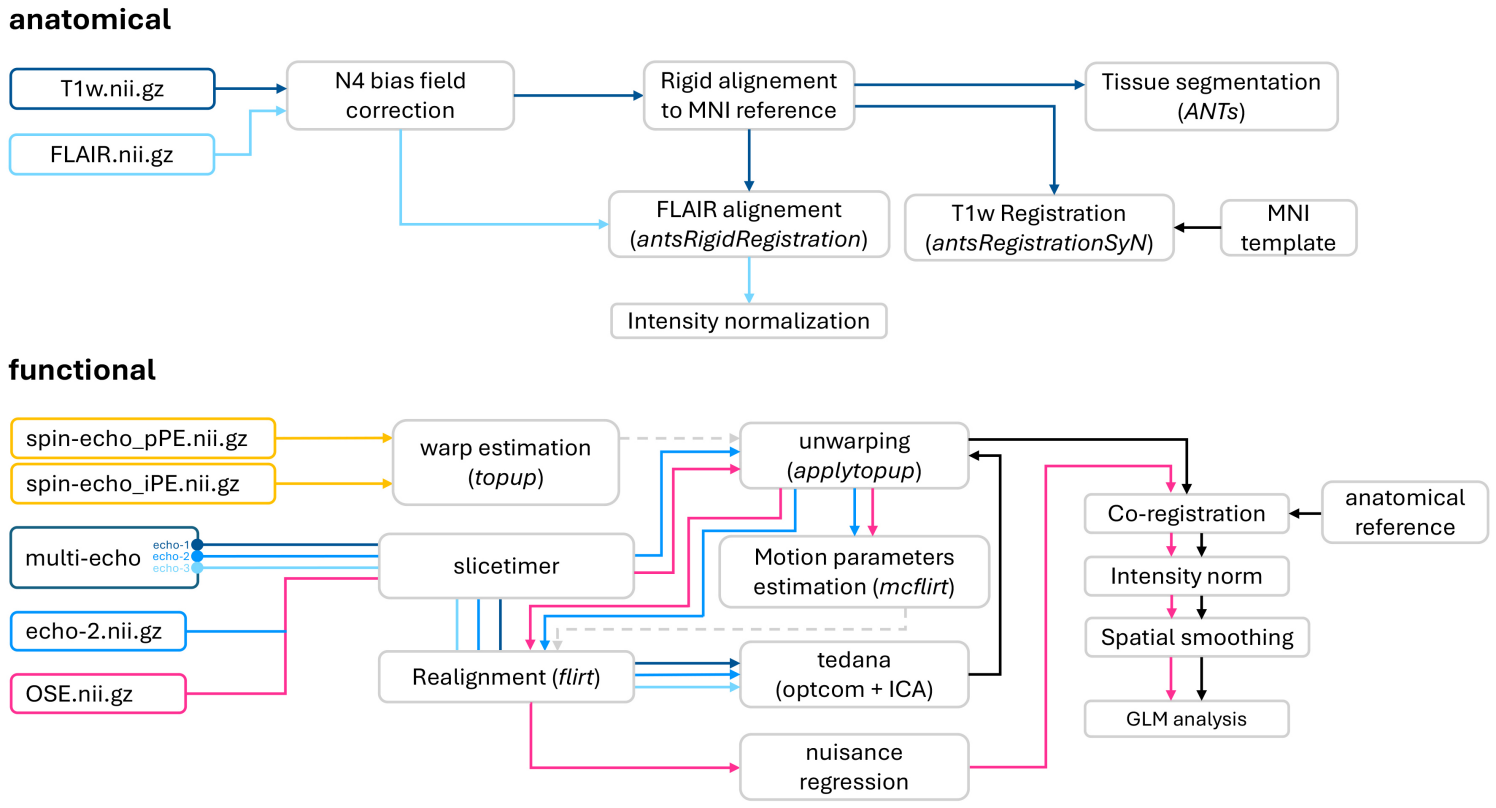
Data preprocessing pipeline. Top row: Flow chart of the anatomical pipeline: structural images undergo bias field correction using the N4 algorithm; the FLAIR image is aligned to the T1-w defined in the MNI space, and intensity normalized. The bottom row reports the flow chart of the fMRI preprocessing pipeline involving slice-timing correction and motion correction (parameters estimated from echo-2 time-series) on each TE. Then, data from the three echoes are combined and denoised (*tedana*), and unwarped to correct for spatial distortions. The single-echo pipeline (applied to OSE and echo-2 time-series) runs slice-timing, spatial distortion, and motion corrections, and regression of signal components of no interest (i.e., white matter and CSF signals). The final steps of both fMRI pipelines are registration to the anatomical image, intensity normalization, and spatial smoothing.

The basic anatomical and functional preprocessing pipeline applied to all data is depicted in Figure 2.

#### 2.4.1 Anatomical MRI

The T1-w anatomical image underwent in-house processing which includes brain extraction, correction for bias field intensity artifacts using the N4 algorithm (Tustison et al., 2010), rigid realignment to the MNI space, and a six tissues segmentation (i.e., cortical GM, WM, deep GM, CSF, stem, cerebellum) (Avants et al., 2011). Finally, the non-linear transformation from the subject T1-w to the MNI template was computed using ANTs (v2.2.0) (Avants et al., 2014) and an ROI parcellation of the cortical GM was defined according to the Yan homotopic parcellation (Yan et al., 2023) with N=200 parcels matched to the Yeo2011 17 networks cortical organization (Yeo et al., 2011).

The FLAIR structural image was corrected using N4 bias field correction, rigidly registered to the T1-w image and intensity normalized. The corrected FLAIR image is used as a reference for the functional MRI analysis (Fig. 2, upper panel).

#### 2.4.2 Functional MRI: single-echo preprocessing pipeline

Spin-echo images were used to estimate the spatial distortions induced by B0 field inhomogeneities using a method similar to that described in Andersson et al. (2003) as implemented in FSL (*topup*; Smith et al., 2004). BOLD fMRI time-series were preprocessed following a standard preprocessing stream. Steps included slice-timing correction (FSL’s *slicetimer*), unwarping to correct for spatial distortions using the B0 map estimated from the spin-echo images (FSL’s *applytopup*; Andersson et al., 2003; Smith et al., 2004), motion correction with a frame-by-frame rigid-body realignment to the middle volume of each run (FSL’s *mcflirt;* Jenkinson et al., 2002), registration to the anatomical image of the subject (Avants et al., 2014) sampled at 2 mm isotropic with an intermediate registration step to the spin-echo image to exploit the higher resolution, intensity normalization, and spatial smoothing with a 2 mm FWHM smoothing kernel (Fig. 2, lower panel, pink pipeline).

This pipeline was applied to the acquired OSE-fMRI data and to the second collected echo (i.e., echo-2; TE = 40 ms) from the acquired ME-fMRI data as to best match the TE to the T2* value of GM (Heunis et al., 2021) and to replicate the comparisons performed in other studies (Gilmore et al., 2022; Heunis et al., 2021).

#### 2.4.3 Functional MRI: multi-echo preprocessing pipeline

ME-fMRI data preprocessing pipeline was constructed upon *tedana*’s (TE-Dependent ANAlysis) python library (https://doi.org/10.5281/zenodo.1250561) and recommendations (https://tedana.readthedocs.io). Spin-echo images were used to estimate the spatial distortions induced by B0 field inhomogeneities using a method similar to that described in Andersson et al. (2003) as implemented in FSL (*topup*; Smith et al., 2004). Each TE time-series underwent slice-timing (FSL’s *slicetimer*), distortion (FSL’s *applywarp*), and realignment (FSL’s *flirt*; Jenkinson et al., 2002) correction steps separately. However, motion parameters were estimated from the echo-2 time-series and the resulting transformation was applied to all echoes of the ME series. Subsequently, data from the three echoes were combined and denoised to remove additional noise using *tedana* (version v0.0.12; https://doi.org/10.5281/zenodo.1250561) (ME-denoised) and unwarped using the previously computed spatial transformation from FSL’s *topup*. Finally, data were registered to the subject anatomical images sampled at 2 mm isotropic resolution, intensity normalized, and spatially smoothed with a 2 mm Gaussian kernel. Transformations to the subject anatomical were computed using the corrected echo-2 mean image.

### 2.5 General linear models

To determine task-dependent activations, activation maps were computed for each dataset (OSE, echo-2, and ME-denoised) using the GLM (General Linear Model) implemented in SPM12 (https://www.fil.ion.ucl.ac.uk/spm/), modeling the effects related to unpracticed reading.

First-level analysis was performed using the default SPM configuration, with the canonical HRF and the high-pass filter of 1/128 Hz. Effects of motion were controlled for in all tasks by including six motion parameters (translation and rotation) and outliers computed with ART (artifact detection tools; https://www.nitrc.org/projects/artifact_detect/) in each subject’s GLM. Subsequently, different contrast maps (see list in Table 2) were computed to describe the activations induced by degraded word reading *versus* the undistorted printed word (used as a baseline), and the different activation intensity induced by the increasing degradation level (i.e., comparing different levels of the same spatial transformation). Most subjects did not exhibit significant activation in response to changes in the contrast percentage of the printed word stimuli. Therefore, we decided to exclude this manipulation from further consideration.

**Table 2.** Contrast maps computed in the analysis of the activation patterns.

Contrast
Mirroring left-right (LR) > baseline
Mirroring upside-down (UD) > baseline
Mirroring LRUD > baseline
Rotation 45° & -45° > baseline
Rotation 15° & -15° > baseline
Rotation 30° & -30° > baseline
Spacing 2 > baseline
Spacing 4 > baseline
Spacing 6 > baseline

Predictors (i.e., SPM betas) and contrast maps of each subject were moved to the MNI space for the second-level analysis using the non-linear transformations computed during the preprocessing of the anatomical images. Population-level main effects of task activations were evaluated using the obtained individual contrast maps in SPM with statistical significance thresholds set to p < 0.001 at the voxel level and p < 0.05 family-wise error (FWE) corrected at the cluster level (p_FWEc_).

### 2.6 Dataset comparisons

We performed multi-level comparisons of the different datasets (i.e., OSE, ME-denoised, echo-2) to fully characterize the impact of the different acquisition protocols.

#### 2.6.1 Time-series comparison

Preprocessed images were compared before the SPM analysis computing the *temporal signal-to-noise ratio (tSNR)*, defined as the ratio between the time-series mean and its standard deviation (Parrish et al., 2000). A higher tSNR indicates a more robust dataset against noise sources. We evaluated the tSNR for each voxel and then computed its average in the cortical GM across runs for each subject. For the ME-fMRI, the tSNR was computed on the BOLD denoised time-series.

#### 2.6.2 Subject-level comparison

For each subject and for each contrast, we derived the activation/difference maps by setting a significant threshold of p < 0.001 at the voxel level (p_UNC-v_) and p < 0.05 family-wise error corrected at the cluster level (p_FWEc_). We characterized the subject-level performances by computing different indices.

##### 2.6.2.1 Activation extent

For each contrast, we computed the volume extension of the significant voxels in the cortical GM and their percentage with respect to the total active voxels (i.e., in all brain tissues).

##### 2.6.2.2 Activation magnitude

The T-statistic of active clusters was computed as a measure of the activation magnitude to compare their strength. For each significant cluster, we computed the 80 percentile of the T-values distribution in the cortical GM and selected, for each contrast, the maximum value.

##### 2.6.2.3 Sensitivity metrics

Sensitivity was evaluated in terms of (a) the number of contrasts with at least one significant cluster and (b) the number of significant clusters for each contrast.

For each performance index, a repeated measure ANOVA was performed to investigate the impact of the acquisition protocol. Furthermore, a subsequent post-hoc pairwise comparison analysis was performed to identify significant differences among the different datasets.

#### 2.6.3 fMRI reliability

Reliability in fMRI refers to the consistency of measured brain activity patterns across repeated experimental runs. The intra-class correlation coefficient (ICC) was used to assess the reliability of ratings (i.e., the degree of agreement between repeated measurements) by comparing the variability of different ratings of the same subject with the total variation across all ratings and all subjects. The ICC is typically defined as the proportion of total measured variance (variability between people, sessions, etc.) that can be attributed to variability between people: $ICC=\frac{\sigma_{\;\;\;\;\;\;subject}^{2}}{\sigma_{\;\;\;\;\;total}^{2}}$ (Noble et al., 2021). Typically, a summary statistic (i.e., mean from the contrast maps *T*-values) for each subject is derived for each ROI and experiment run, and ICCs are then computed for these values (Caceres et al., 2009). In our analysis, mean *T*-value from regions provided by the Yan homotopic parcellation (Yan et al., 2023) was extracted from each subject’s contrast maps. For each ROI, ICC(3,1) was computed to assess consistency across runs. Specifically, ICC(3,1) is defined as

$$ICC\left(3,1\right)=\frac{MSB-MSE}{MSB+\left(k-1\right)MSE},$$ (1)

modeling a two-way fixed effects model to calculate the reliability of raters (i.e., runs) as individuals. In these models, the total sum of squares is split into subject (MSB) and error (MSE) sums of squares, and k is the number of repeated runs. For ICCs, the following criteria as developed by Cicchetti and Sparrow (1981) were applied for interpretation: poor (below 0.4), fair (0.4-0.59), good (0.6-0.74), and excellent (≥0.75).

#### 2.6.4 Group-level comparison

We focus these analyses on the comparison between OSE-fMRI and ME-fMRI data as in the subject-level analysis they proved to be significantly better than echo-2 data.

We investigated differences between the two datasets at a group level evaluating the following performance features.

##### 2.6.4.1 Activation analysis

We performed population-level voxel-wise analysis to verify whether the considered task elicited the recruitment of the dorsal pathway.

##### 2.6.4.2 Power analysis

To investigate the impact of the acquisition protocol in a hypothetical group experiment, we performed a statistical power analysis for a two-sided *t*-test for two independent samples to estimate the sample size needed to detect a statistically significant effect in the contrast images. The significance level, that is, the probability of incorrectly rejecting the null hypothesis when it is true (Type I error), was adjusted to 0.05 using the Bonferroni correction among ROIs. The power of the test, that is, the probability of correctly rejecting the null hypothesis when the alternative hypothesis is true (1 - Type II error), was set to 0.8. The sample size estimates were estimated using a two-sample *t*-test from 200 ROIs identified by the Yan homotopic parcellation.

##### 2.6.4.3 Paired t-test analysis of fMRI sequence effects

To compare the effects of the two acquisition sequences on the task-related activations, a second-level analysis was performed using a paired *t*-test. First-level contrast images of the reading task were parceled to extract a mean contrast value for each ROI of the atlas. These values were then entered into a paired *t*-test to evaluate within-subject differences between the sequences, accounting for inter-subject variability. The significance level was adjusted to 0.001 using the Bonferroni correction among ROIs.

## 3 Results

### 3.1 Dataset comparison

#### 3.1.1 Time-series comparison

**Fig. 3.**
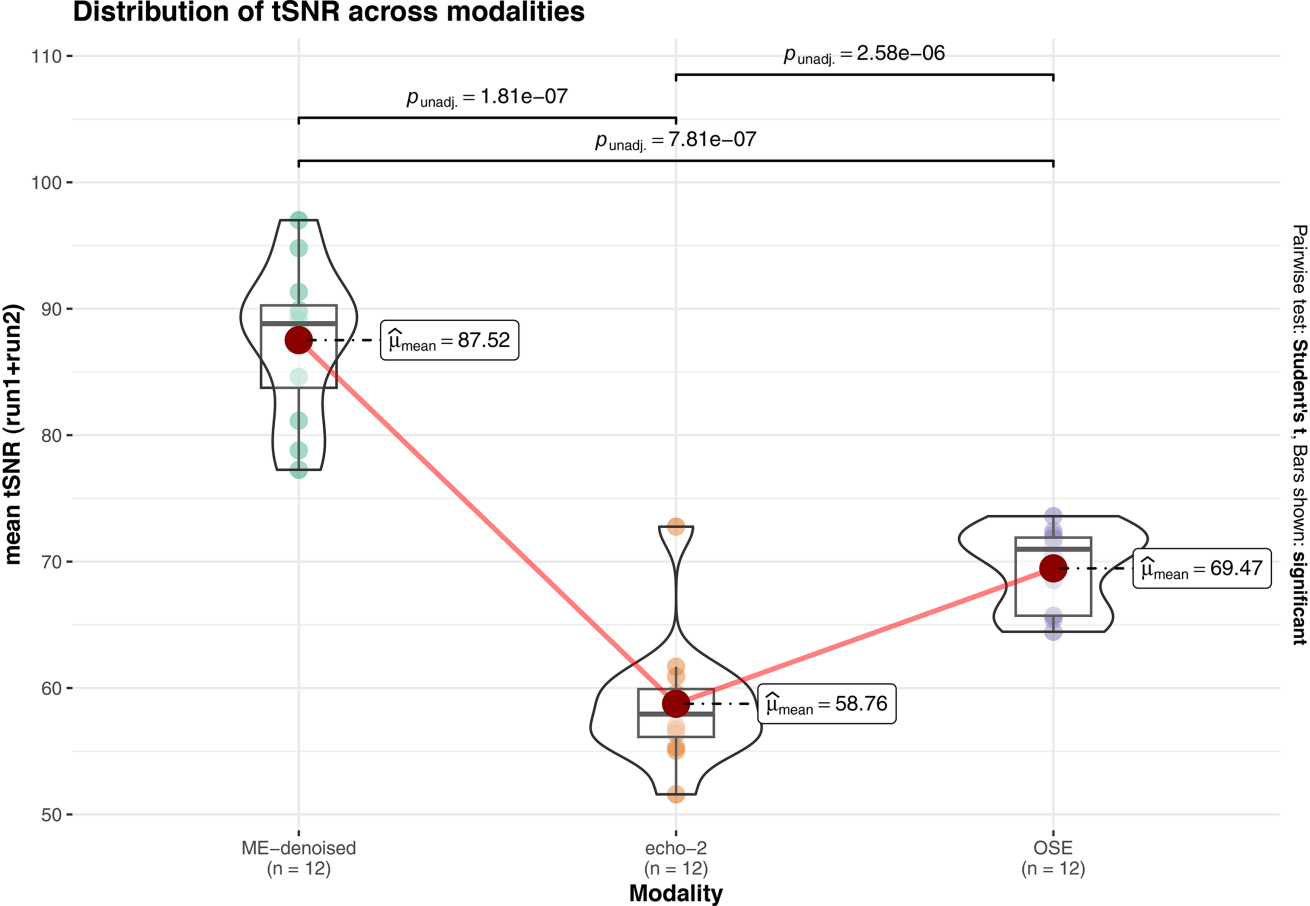
Box/violin plots for repeated measures comparisons of mean GM tSNR over all participants and runs. Values reflect the time-series mean divided by its standard deviation. Points represent the mean tSNR over the cortical GM tissue of each subject. From left to right: ME-denoised: multi-echo combined and denoised, echo-2: middle echo extracted from multi-echo sequence, OSE: optimized single echo.

Figure 3 reports the comparison of the tSNR in the cortical GM among fMRI acquisition sequences. Fisher’s repeated measures one-way ANOVA revealed a significant effect of the acquisition sequence (p < 0.001), and the subsequent post hoc pairwise *t*-tests identified a significantly larger tSNR in both OSE and ME-denoised with respect to echo-2, in ME-denoised with respect to OSE. Spatial patterns of tSNR distribution (Supplementary Fig. S1) confirm that multi-echo sequences enhance BOLD sensitivity in regions prone to signal dropout in conventional single-echo acquisitions.

#### 3.1.2 Subject-level comparison

##### 3.1.2.1 Activation extent

**Fig. 4.**
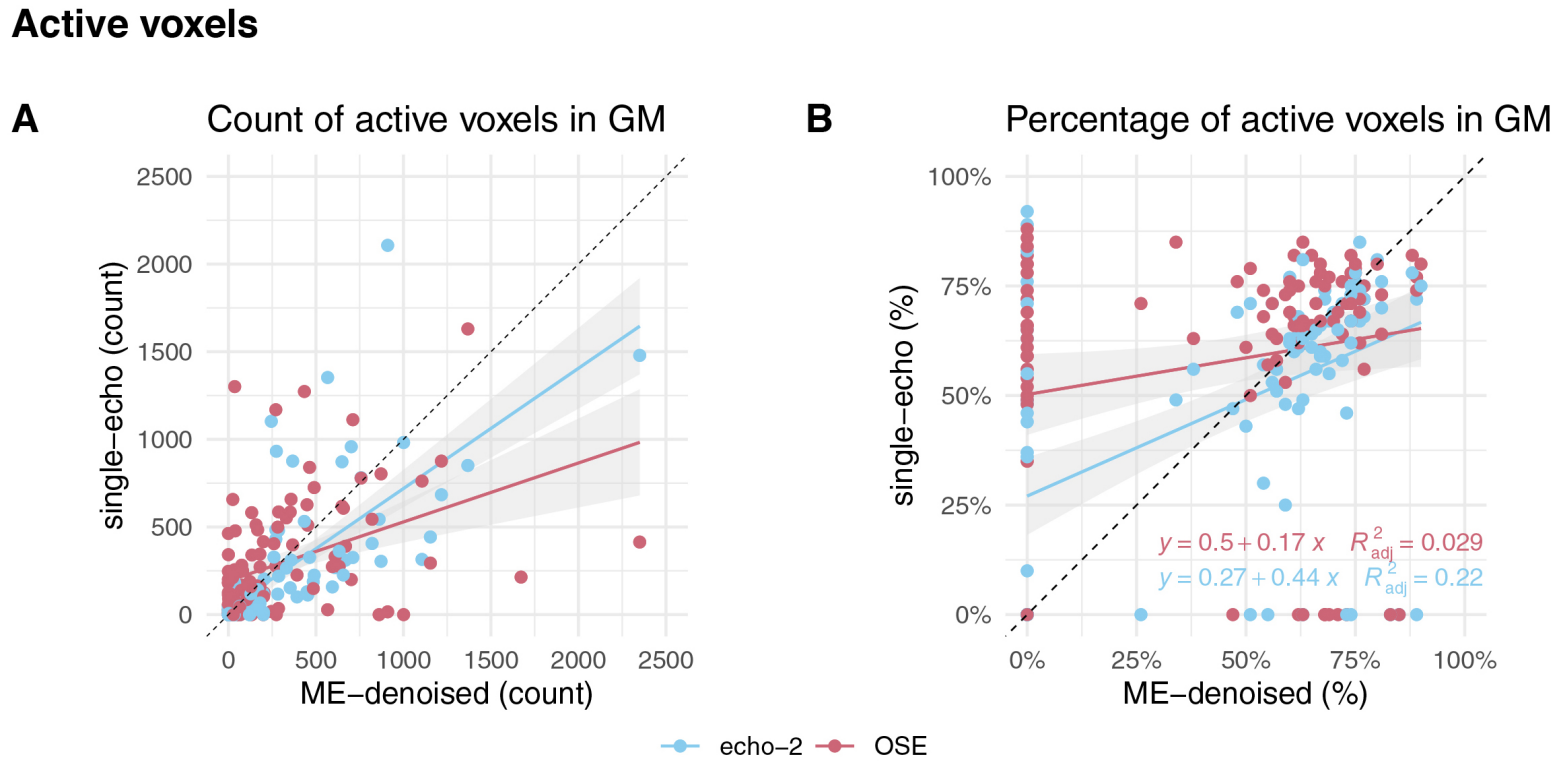
Plot of the GM activation extent comparison. Panel (A) reports the number of active voxels restricted in GM over all participants and contrasts, panel (B) shows the percentage of active voxels in GM with respect to the total activation volume (i.e. including all brain tissues).

When considering the voxel-wise activation maps computed for each subject, we observed a larger activation extent for ME-fMRI data with respect to OSE-fMRI data and a similar extension pattern between ME-fMRI and echo-2 data (Fig. 4A). However, OSE-fMRI activation pattern seems to overlap more to the cortical GM with respect to ME-fMRI, while ME-fMRI and echo-2 data show a similar GM overlap ratio across the different experiments (Fig. 4B).

##### 3.1.2.2 Activation magnitude

**Fig. 5.**
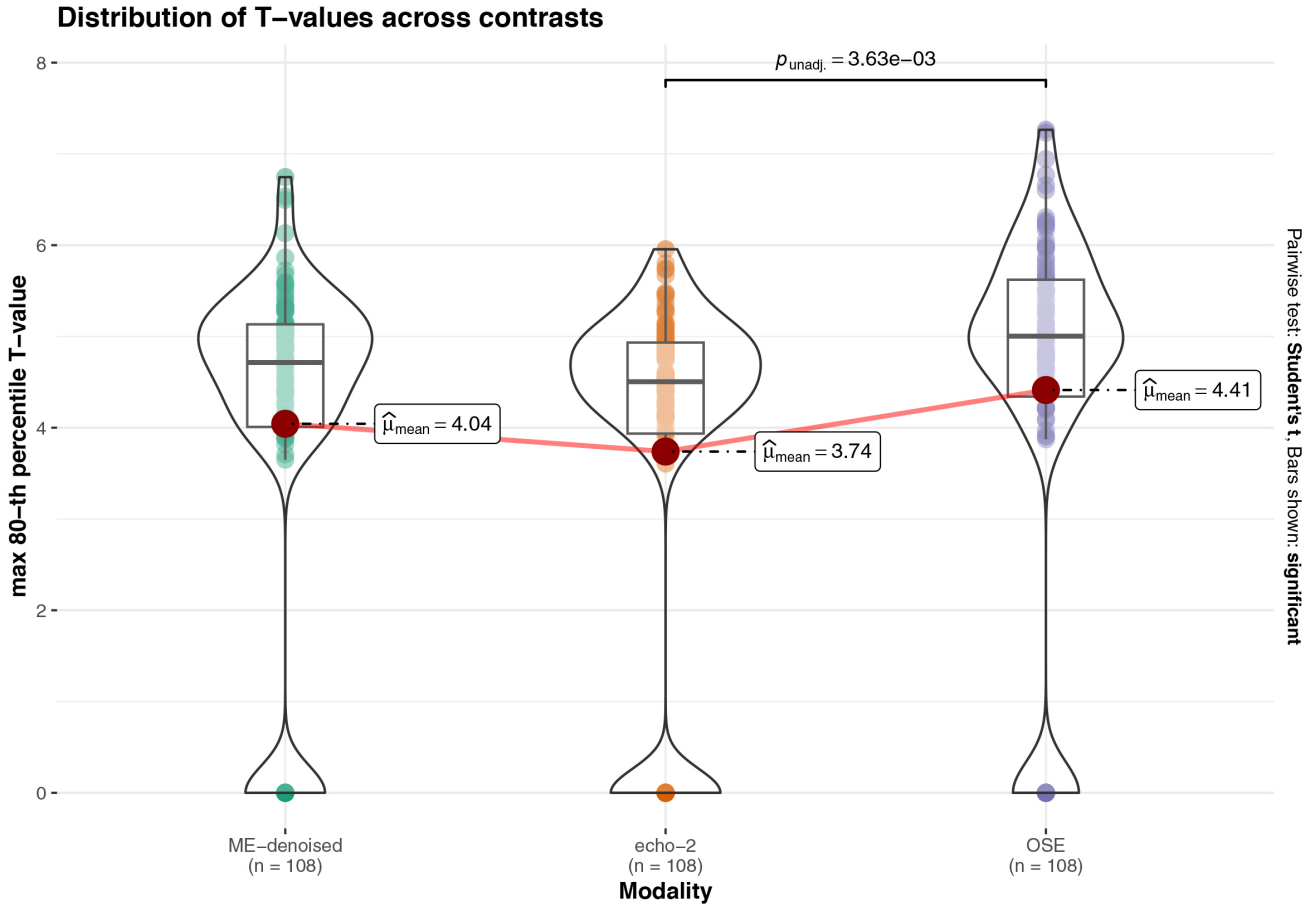
Boxplot of T-statistics comparison. The chart reports the maximum of the T-statistics 80-percentile values from the clusters related to the task-based contrast maps resulting from participant-level GLM analysis. We superimposed the boxplot representation with the real data (colored dots). Zero values represent subjects and contrasts with no active clusters.

OSE-fMRI data are also characterized by a significantly larger activation magnitude in the cortical GM with respect to both and echo-2 data (Fig. 5), as confirmed by the statistical comparisons. While no differences were identified between ME-fMRI and echo-2 data.

##### 3.1.2.3 Sensitivity metrics

**Fig. 6.**
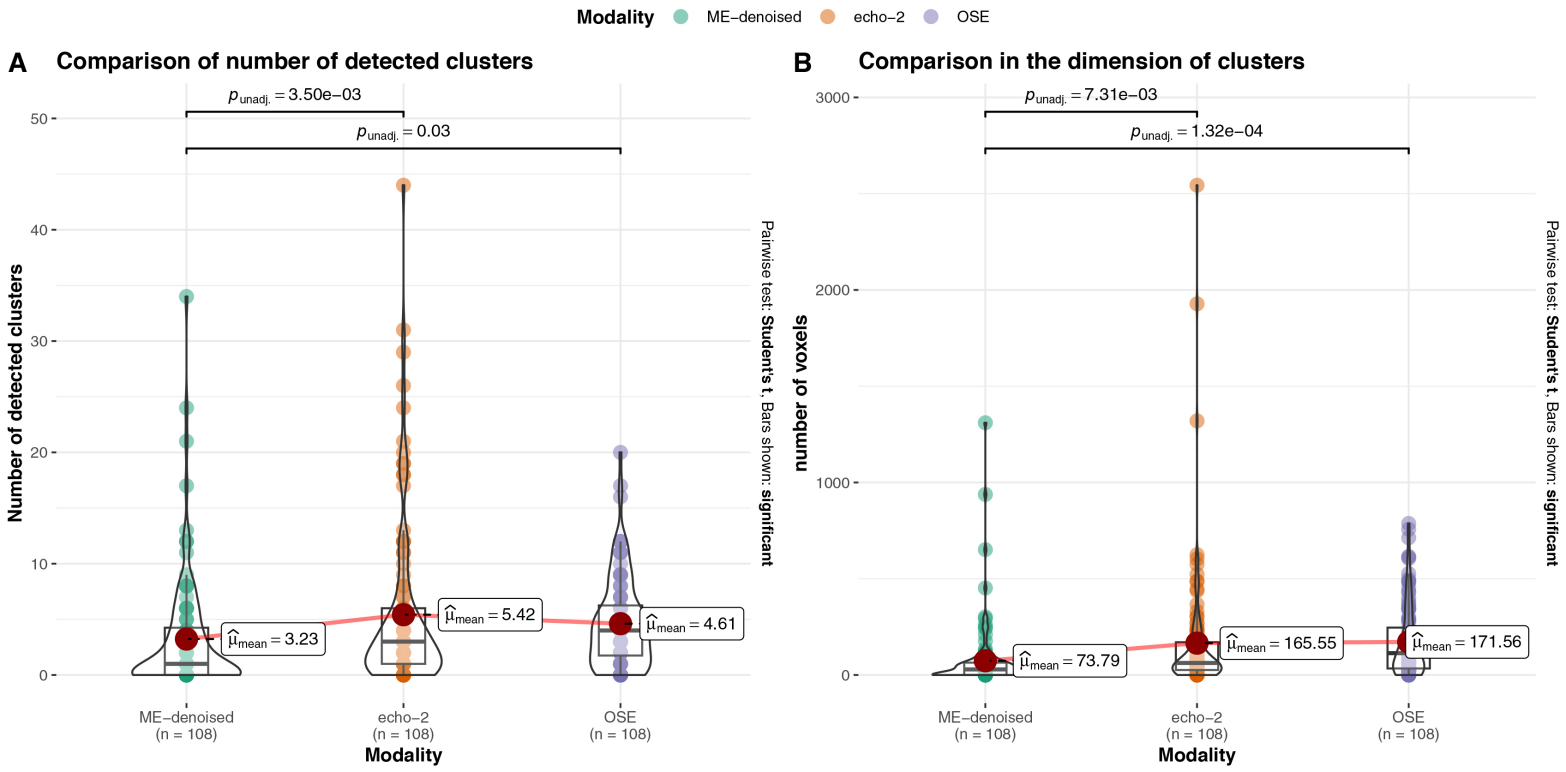
Boxplots of sensitivity metrics over all participants and contrasts analyzed. Panel (A) reports the relative number of clusters and Panel (B) shows the distribution of the clusters’ dimensions. We superimposed the boxplot representation with the real data (colored dots).

The sensitivity analysis showed that the echo-2 data identified a larger number of active clusters than ME-fMRI and OSE-fMRI data (Fig. 6A), but also that those clusters are in general smaller than those identified from the other data (Fig. 6B), suggesting that echo-2 data lead to more fragmented activation maps than ME-fMRI and OSE-fMRI data. In contrast, no differences were highlighted from the sensitivity analysis between ME-fMRI and OSE-fMRI data.

#### 3.1.3 fMRI reliability

A ROI-wise ICC brain map was obtained by applying Eq. (1) to the subject contrast images. Thresholding of *T*-values corresponding to p < 0.001 at ROI level was used to define the activation networks evoked by the task. Higher ICCs were observed in the ME-denoised sequence than in the OSE sequence, ranging from poor to good depending on the contrast (Table 3).

**Table 3.** Reliability of task-fMRI contrasts.

	ME-denoised	OSE
fMRI contrast	median [P5 - P95]	median [P5 - P95]
Mirroring LR	0.524 [0.463 - 0.667]	0.122 [-0.084 - 0.408]
Mirroring UD	0.361 [0.035 - 0.519]	0.257 [0.01 - 0.485]
Mirroring LRUD	0.275 [0.118 - 0.457]	-0.004 [-0.078 - 0.102]
Rotation 45° & -45°	0.382 [0.138 - 0.387]	0.274 [-0.119 - 0.48]
Rotation 15° & -15°	-	0.105 [0.013 - 0.173]
Rotation 30° & -30°	-	0.425 [0.024 - 0.713]
Spacing 2	-0.067 [-0.318 - 0.185]	-0.014 [-0.412 - 0.094]
Spacing 4	0.471 [0.264 - 0.597]	0.32 [0.142 - 0.415]
Spacing 6	0.324 [0.079 - 0.611]	0.021 [-0.44 - 0.594]

The table reports the median [5th percentile - 95th percentile] of ROI-wise ICCs ME-denoised and OSE sequences computed with Eq. (1).

**Fig. 7.**
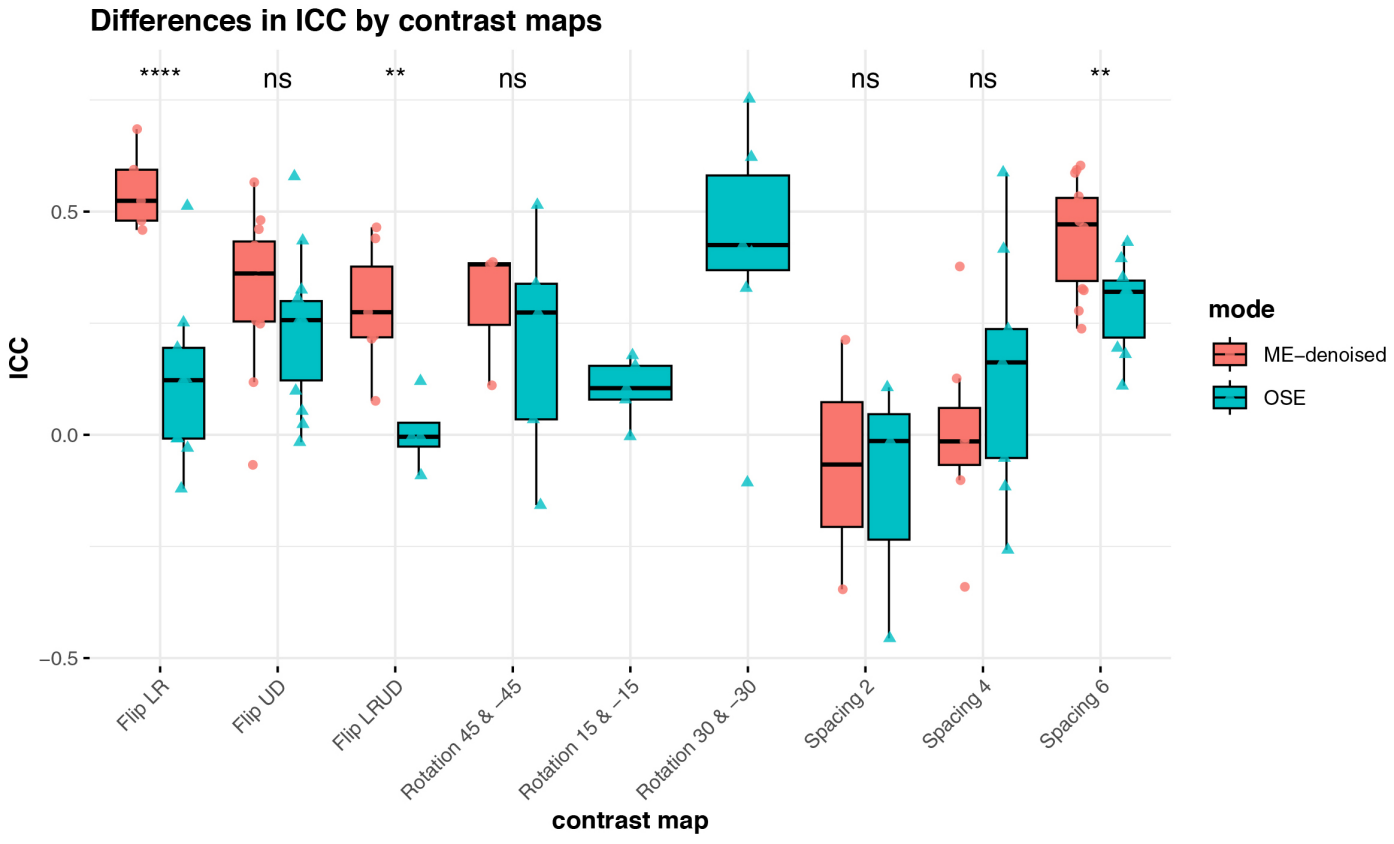
Boxplot of reliability score of task-fMRI contrasts. The figure shows the ROI-wise ICCs of ME-denoised, and OSE sequences computed by Eq. (1). Each contrast ICC distribution was tested for differences between modalities with a pairwise *t*-test (ns: p > 0.05; **p < 0.01; ****p < 0.0001).

The contrasts showing a significant difference between modalities favor the multi-echo approach (Fig. 7).

#### 3.1.4 Group-level comparison

##### 3.1.4.1 Activation analysis

**Fig. 8.**
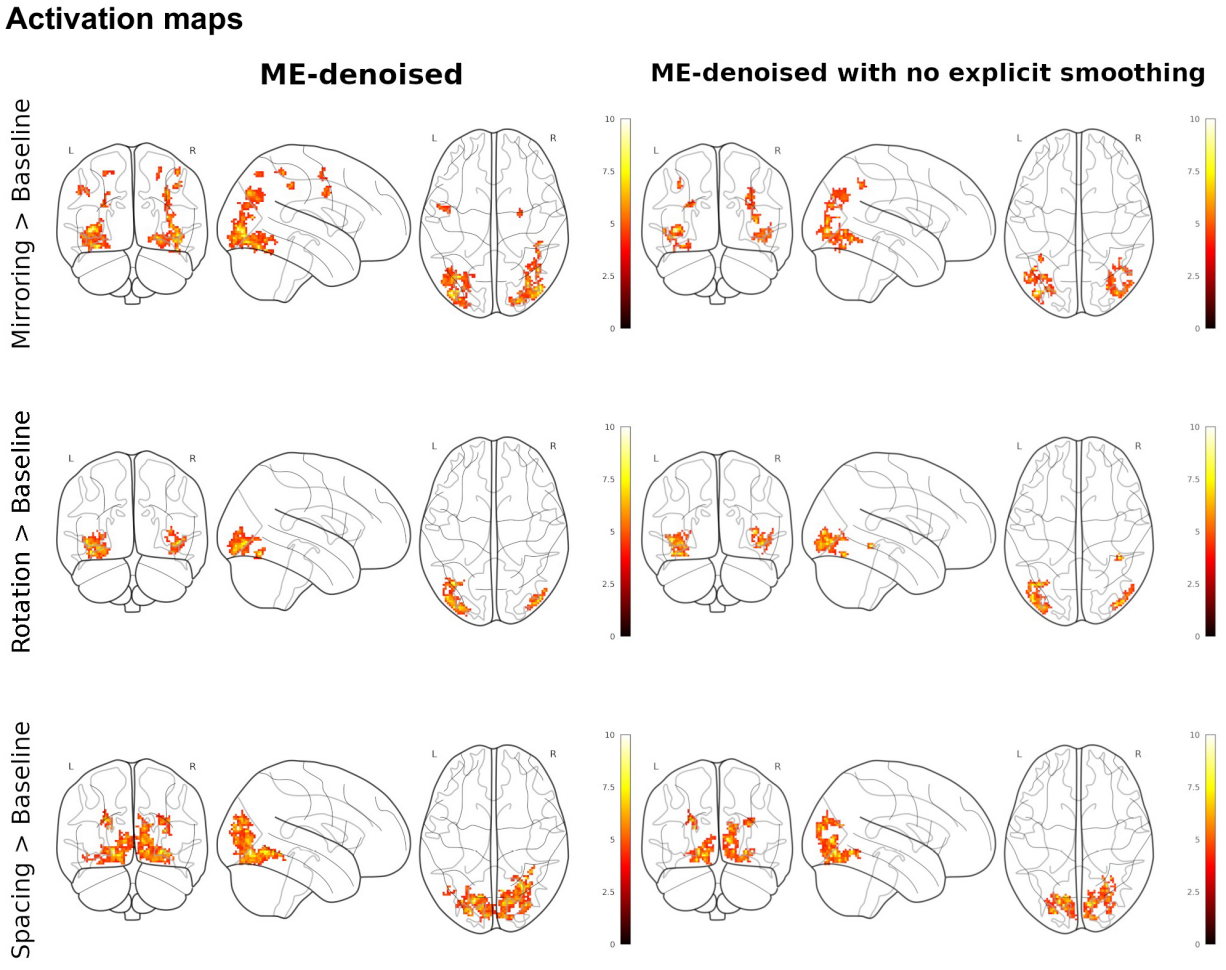
Group-level analysis of the reading task. Activations are reported on a glass brain at p < 0.001 (voxel-level) and p < 0.05 FWE corrected (cluster-level) for the Mirroring LR vs. baseline (top row), Rotation ± 45° vs. baseline (mid row) and Spacing 6 vs. baseline (bottom row) contrasts.

Figure 8 shows the group-level maps of activation across runs for multi- and single-echo under an FWE correction at a cluster level, for three main contrasts computed: Mirroring (UD) > Baseline, Rotation (± 45°) > Baseline, and Spacing (6) > Baseline. In our analysis, the cluster size threshold corresponding to a corrected p < 0.05 was determined using cluster-level family-wise error (FWE) correction as implemented in SPM12. For the reported results, a voxel-wise threshold of p < 0.001 (uncorrected) was applied, and clusters exceeding a minimum extent of voxels were considered significant at p < 0.05 FWE corrected. See Table S2 in Supplementary materials for the cluster size threshold of the contrast analyzed.

Both OSE-fMRI and ME-fMRI reveal activation maps visually coherent with each other across all three contrasts examined. As such, the regions of brain activation identified by OSE-fMRI and ME-fMRI are largely consistent in terms of location and extent. The visual compatibility across these contrasts suggests that both acquisition sequences are effective in reliably capturing the same neural activity patterns.

##### 3.1.4.2 Power analysis

**Fig. 9.**
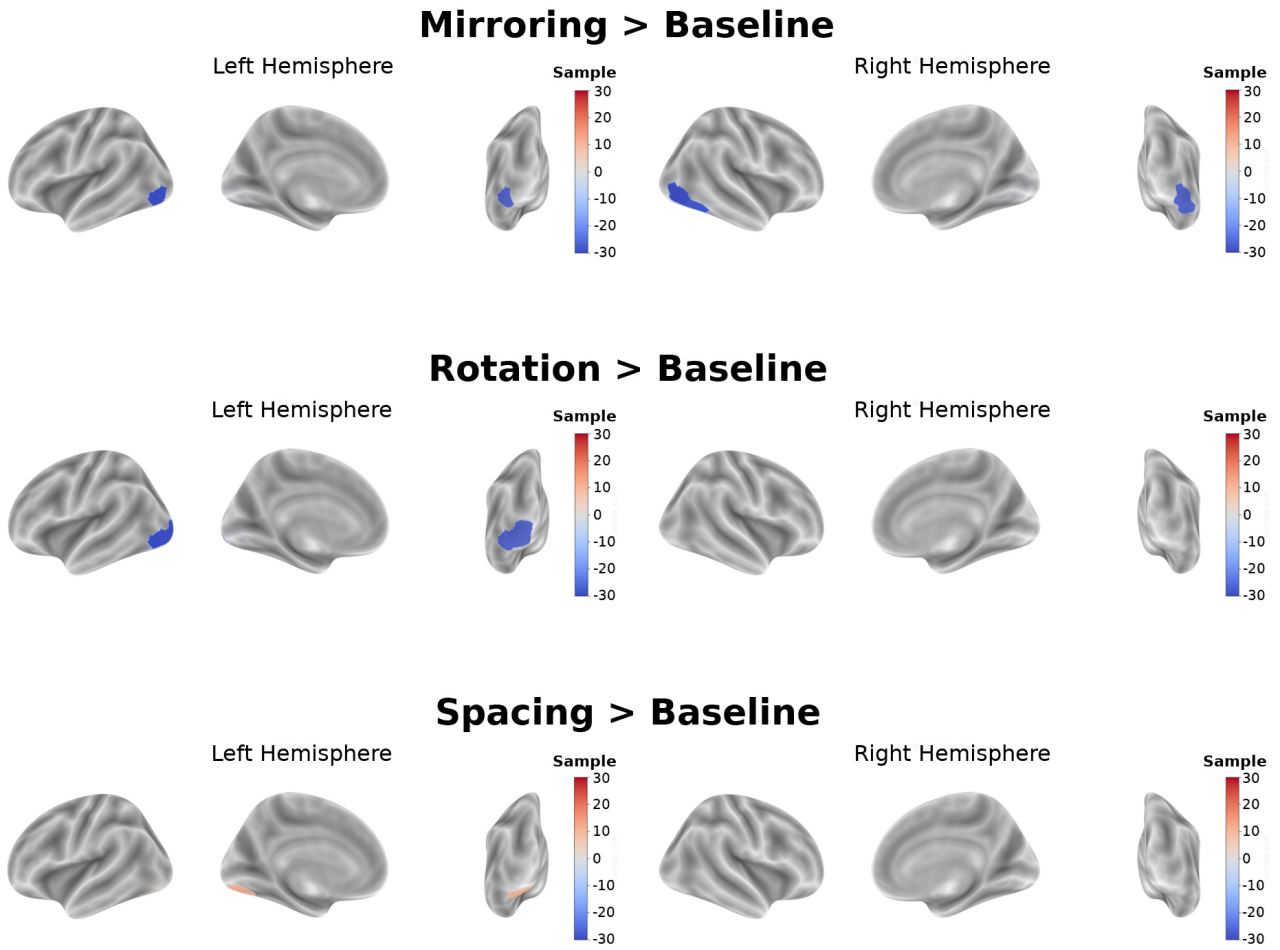
ME-denoised and OSE comparison in the sample size estimates. Surface plotting of the ROI-wise difference between OSE and ME-denoised in sample sizes required by the three main contrasts (*Mirroring UD* *>* *Baseline, Rotation* *±* *45°* *>* *Baseline, Spacing 6* *>* *Baseline*) to get statistically relevant differences in activation with a power of 0.8, in the common active regions. A positive (red) value indicates that the ME-denoised sequence requires a smaller sample size to capture differences; conversely, a negative (blue) value signifies that the ME-denoised sequence requires a larger sample size.

The sample size needed to get a statistically significant effect in the contrast images is summarized in Table 4. Based on previous findings (Mascheretti et al., 2024), the difference in the percentage of the mean of two groups performing a visual task ranges between 28% and 61%. Therefore, we assumed this difference to be 45%. To spatially investigate any differences between the two analyzed sequences, we surface plotted the difference in sample sizes estimated between OSE and ME-denoised on the three main contrast images (Fig. 9).

**Table 4.** Summary of ME-vs-OSE in the sample size estimates.

	ME-denoised		OSE	
fMRI contrast	Mean (SD)	Active regions	Mean (SD)	Active regions
Mirroring LR	83	1	59 (19)	7
Mirroring UD	81 (10)	6	57 (25)	9
Mirroring LRUD	80 (26)	2	77 (20)	4
Rotation 45° & -45°	84 (4)	3	57 (22)	5
Rotation 15° & -15°	104	1	61 (17)	4
Rotation 30° & -30°	-	0	57 (16)	5
Spacing 2	70	1	69 (5)	2
Spacing 4	83 (16)	2	100	1
Spacing 6	89 (15)	5	77 (4)	4

Mean and standard deviation across ROIs of the sample sizes estimated to get statistically relevant differences between groups in activation for multi-echo and single-echo sequences.

##### 3.1.4.3 Paired t-test analysis of fMRI sequence effects

The paired *t*-test revealed significant differences in task-related activation of *Rotation* *±* *45°* *>* *Baseline* contrast between the two acquisition sequences in the visual system of the left Striate Cortex (Visual B network and temporal-parietal network of the Yeo2011 17 networks (Yeo et al., 2011)). These differences were localized using p < 0.001, Bonferroni corrected. Increased activation was observed in the *17networks_LH_VisPeri_Striate* for ME-denoised compared with OSE (*t*(*n*-1) = 5.59, p_corrected_ < 0.05).

## 4 Discussion

In this study, we explored the practical benefits of a potentially more physiologically reliable fMRI acquisition sequence (i.e., ME) during a reading task over conventional single-echo fMRI acquisitions. In the context of the current investigation, the advantages of ME acquisition technique were quantified by comparing three different datasets, that is, the data acquired with an OSE-fMRI sequence, those acquired with an ME-fMRI sequence, and the echo-2 time-series extracted from the ME-fMRI data itself. The efficacy of the ME approach has been documented elsewhere (Gonzalez-Castillo et al., 2016), where its advantages were derived from the comparison against a single-echo extracted from the ME acquisition (i.e., echo-2). Given that the ME-fMRI *vs.* echo-2 comparison was performed as a replication study. Instead, in this study, we sought to investigate a more realistic scenario in which both the ME-fMRI and the OSE acquisitions are optimized for a given experiment. To this end, both acquisitions were tailored to maximize their strengths and meet the experimental constraints: sequences were optimized to maximize temporal resolution and spatial coverage, which is crucial for tasks requiring high temporal fidelity (e.g., event-related designs) and detection of rapid neural events.

In designing the multi-echo fMRI protocol for this study, the choice of TEs (14 ms, 40 ms, 66 ms) was guided by a deliberate trade-off between the aforementioned constraints and the performance of the scanner (e.g. duty cycle limits, maximum gradient strength, and slew rate). We prioritized a relatively short TR (1650 ms), which was essential for task-based fMRI analysis to maximize the number of samples acquired, thereby increasing the statistical power of our analysis. The choice of TEs (14-40-66 ms) reflects a trade-off between capturing BOLD signal evolution and minimizing signal dropout in regions with short T2 values. While the middle echo time of 40 ms differs from the one employed in previous studies (i.e., ~30-40 ms), it ensures coverage of the peak of BOLD contrast at 3T (Peters et al., 2007). No universally optimal protocol exists for all experimental scenarios; our choices reflect a balance tailored to our study goals and scanner capabilities. Within the constraints of our setup—including fixed multiband and SENSE factors—we selected the most efficient configuration achievable without compromising the image quality. As discussed, optimizing scan parameters always involves trade-offs between image quality, scan duration, and artifact minimization.

The comparison between echo-2 and Multi-Echo (ME-denoised) confirms the findings reported in previous studies (Dipasquale et al., 2017; Gilmore et al., 2022; Gonzalez-Castillo et al., 2016; Heunis et al., 2021), which have consistently highlighted the advantages of the ME approach over single-echo acquisitions. However, it is important to acknowledge that this comparison has inherent limitations. The primary limitation is that it does not allow for definitive conclusions regarding the use of an optimized single-echo acquisition. Instead, it mainly addresses the implications of utilizing part of the ME data (i.e., subsampling the acquired images to retain single-echo data from the signal sampled at several TEs) as opposed to leveraging the full potential of the ME sequence.

In this study, the echo-2 data significantly differ from the OSE data: (i) OSE acquisition achieves a shorter TR, resulting in a greater volume of data being collected within the same experimental duration, (ii) echo-2 data suffer from an MRI signal degradation associated with the spatial resolution, spatial coverage, the TEs, and other constraints related to the inherent complexity of sequence setup in ME acquisitions. Additionally, although our echo-2 (TE=40 ms) is longer than the average echo-2 used in previous studies, it still remains below the typical T2* value for gray matter at 3T (Peters et al., 2007). We selected the second echo for comparison purposes to align with the standard practice in the literature. However, given that the third echo of most previous studies better matches the expected T2* value, future comparison might benefit from considering it as a more appropriate single-echo reference.

The comparison presented here, between OSE and ME sequences, represents a significant contribution to the field, as it (to our knowledge, for the first time) enables a direct evaluation of two fundamentally different acquisition approaches at their respective optimal capabilities. This direct comparison provides valuable insights into the strengths and limitations of each method, offering a more comprehensive understanding of their relative performance and potential applications in various MRI studies. We evaluated an optimized single-echo sequence based on its potential for a more efficient and simplified approach to data acquisition and preprocessing. A single-echo sequence can be tailored to maximize temporal resolution and spatial coverage, which are crucial for certain tasks requiring high temporal fidelity (e.g., event-related designs) and detection of rapid neural events. Each sequence may offer specific advantages depending on the type of experiment and population being studied.

In our analysis, the ME-denoised sequence produced a higher tSNR across the cortical GM tissue, which may indicate a more robust dataset against noise sources, while OSE sequence resulted in activation maps that were more accurately localized inside the GM tissue. This difference in spatial specificity could be partially explained by the larger voxel size used in the ME acquisition, which may increase partial volume effects and lead to spatial blurring of the activation patterns. Importantly, regional heterogeneity in tSNR should be carefully considered when selecting an acquisition sequence, as different brain regions may benefit from different approaches. For example, if a study targets early visual cortex, the higher tSNR achievable with OSE may be preferable; conversely, when focusing on anterior temporal lobe (ATL) or orbitofrontal cortex (OFC), ME acquisition could provide superior signal quality due to its improved tSNR in these regions. These region-specific considerations further highlight the importance of tailoring acquisition choices to the specific anatomical and functional priorities of each study. It should be mentioned that, despite past debates, there is no direct evidence against measuring fMRI activations in white matter, and reports of fMRI activations in white matter continue to increase (Gawryluk et al., 2014). At the subject level, the ICC metrics were computed to assess the reliability of repeated task fMRI runs between the two MRI sequences, favoring the ME-denoised data against OSE in providing more consistent and reproducible measurements. This higher reliability of ME data, however, may come at the cost of reduced sensitivity to task-related activations, suggesting that while ME may be more robust and stable across runs, it might also be less sensitive to subtle task-driven effects. When activations were detected—and ICC computed, values were consistently higher in ME than in OSE, reinforcing its potential for reproducibility in activation-based studies. To further investigate this result, we performed a model-free estimation of the BOLD response using a finite impulse response (FIR) approach, which does not impose a fixed hemodynamic response shape. When using this framework, the difference in ICC values between ME and OSE datasets was substantially reduced (Supplementary Fig. S2). This finding suggests that the canonical HRF model is particularly well suited to fit ME data (see Supplementary Fig. S3), which may contribute to the higher ICC observed under this model. To further evaluate the practical implications of the two MRI sequences, a power analysis was conducted to estimate the sample size required to detect a significant difference in the activation patterns between two hypothetical groups. This comparison provides insights into the relative efficiency of each sequence in identifying group-level effects and informs their potential application in future studies. The OSE sequence demonstrated superior performance in terms of power, as it required a smaller sample size to detect differences across all contrasts evaluated.

Another important consideration in ME fMRI is the potential decoupling between beta estimates and statistical sensitivity. Previous studies (e.g., Gonzalez-Castillo et al., 2016; Gilmore et al., 2022) reported that ME acquisitions often yield reduced beta magnitudes compared with single-echo data, yet improved T-statistics due to superior noise suppression. In our dataset, we observed a similar pattern when comparing ME with echo-2 data, suggesting enhanced sensitivity. However, when the comparison is performed with the OSE acquisition, this trend is no longer observed (see Fig. S4 in Supplementary Materials).

While this study provides insights into the comparative performance of ME and OSE fMRI sequences, it is important to acknowledge some limitations that could influence the interpretation of the results. Preprocessing pipelines were configured according to widely adopted practices in the literature (Aurich et al., 2015; Esteban et al., 2020). However, Mayer et al. (2019) demonstrated that ICA-based denoising methods, while powerful, may inadvertently remove task-related signals—especially in event-related designs—due to partial overlap between task-related variance and components classified as noise. This raises concerns about the potential underestimation of true neural activity in ICA-denoised data. For ME fMRI, activation analysis depends on the denoising technique used in the tedana pipeline, which relies on the differentiation of BOLD from non-BOLD components performed by ICA. Estimates of the signal of interest and noise components depend on the goodness of fit of the data from echoes to an exponential model and can be critical, as it relies on only three data points (i.e., three echoes). As a consequence, the removal of a mixed component (containing noise and non-noise) could result in the partial removal of the BOLD response of interest. One potential direction to mitigate this limitation is to act on the Field Of View (FOV) and reduce the echo spacing to enable shorter TEs, improving the robustness of the exponential T2* fitting. It is, therefore, essential to interpret ME results in light of these methodological caveats, particularly in paradigms where task-related responses may be subtle or temporally sparse.

Furthermore, it is important to highlight that no universally optimized fMRI protocol can be considered “the best for all” experimental scenarios. Each acquisition strategy comes with inherent trade-offs. In our study, the OSE sequence was optimized within specific constraints, such as achieving the shortest possible TR to suit our event-related task design. This means that the resulting protocol may not generalize to all paradigms or research questions. The choice between ME and OSE sequences should thus be driven by the specific priorities of a study—whether that is maximizing temporal resolution, enhancing spatial specificity, improving signal-to-noise ratio, or increasing reliability across sessions.

In conclusion, for an adequate comparison of fMRI acquisition techniques, it is crucial to acquire them independently and at their optimal capabilities; as such, the ME-fMRI acquisition should be compared with an optimized SE-fMRI acquisition. In the current implementation, and on a 3T scanner, the acquisition of multi-echo fMRI (ME-fMRI) does not consistently outperform an optimized single-echo fMRI (OSE-fMRI). ME-fMRI appears to offer greater reliability at the subject level, while OSE-fMRI provides stronger statistical power at the group level. Further research and technical refinements will be essential to clarify the conditions under which one sequence may be preferred over the other.

## Supplementary Material

Supplementary Material

## Data Availability

The data that support the findings of this study are available from the corresponding author, D.P., upon reasonable request. The data are not publicly available due to privacy or ethical restrictions.
