# Supplementary material for: Multi-echo versus single-echo EPI sequences for task-fMRI: A comparative study
Source: Imaging Neurosci (Camb). 2025 Jul 28;3:IMAG.a.94. doi: 10.1162/IMAG.a.94 (PMC12330833; doi:10.1162/IMAG.a.94)
Supplement: Supplementary Material [file IMAG.a.94_supp.pdf]

Supplementary Materials

Figure S1. Voxelwise tSNR maps for ME, echo-2 and OSE sequences.
Comparison of signal quality between single-echo and multi-echo acquisitions. The multi-echo approach recovers signal in areas affected by susceptibility-induced dropout, demonstrating improved BOLD sensitivity in regions typically challenging for conventional single-echo fMRI.

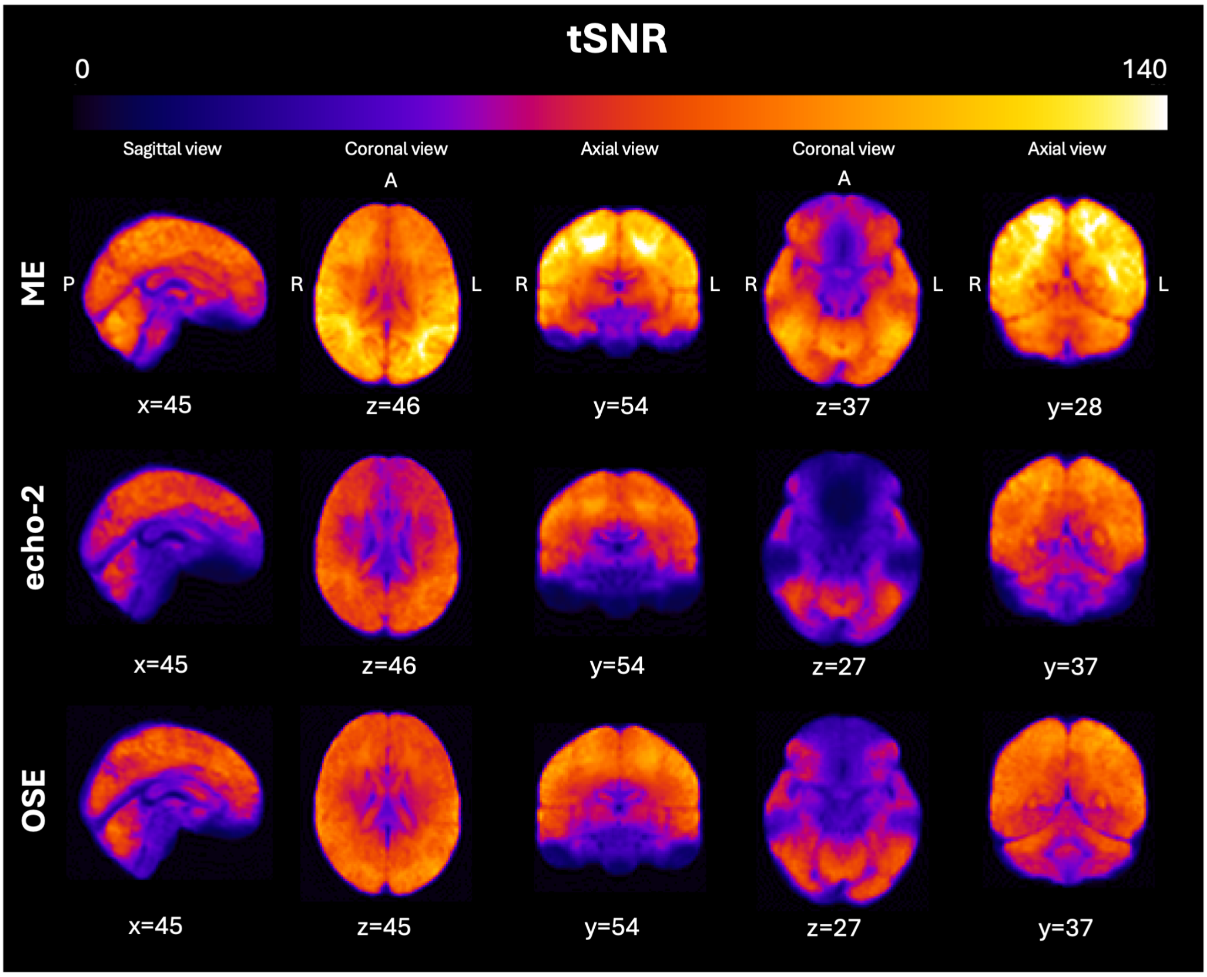

Figure S2. Boxplot of reliability score of task-fMRI FIR contrasts. The figure shows the ROI-wise ICCs of ME-denoised, and OSE sequences when a FIR analysis is performed. Each contrast ICC distribution was tested for differences between modalities with a pairwise t-test (ns: $p>0.05$, *: $p<0.05$, **: $p<0.01$, ***: $p<0.001$, ****: $p<0.0001$).

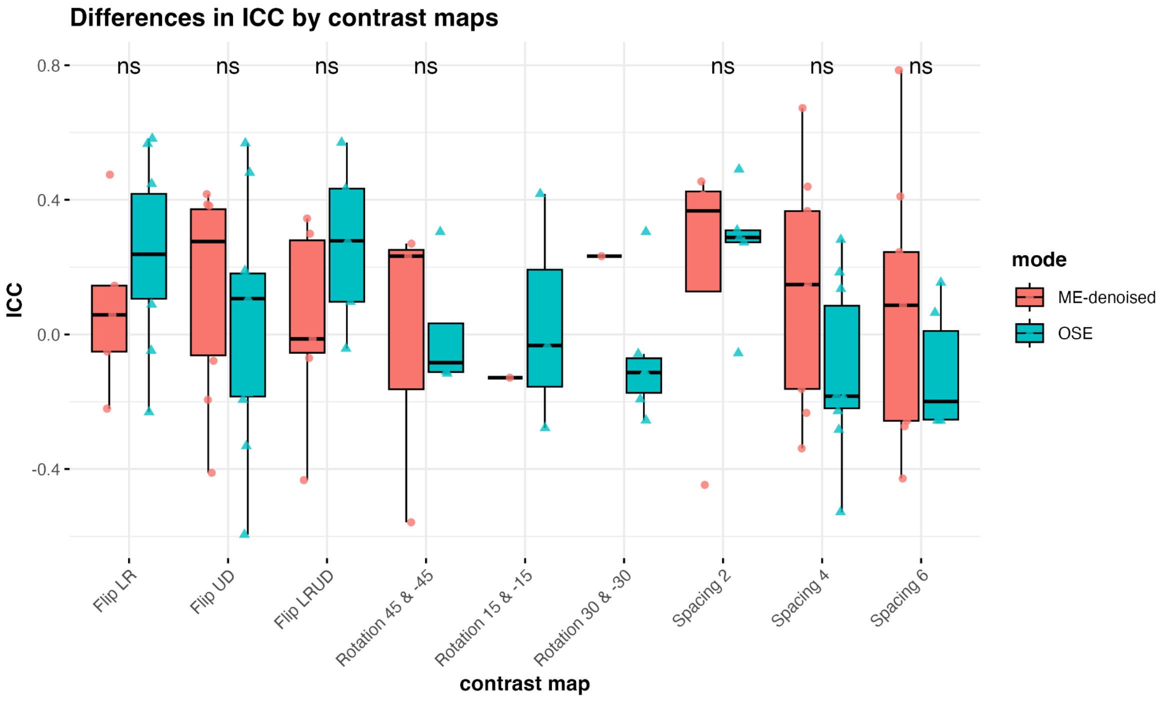

Figure S3. Estimated BOLD signal comparison of FIR and HRF analyses. Comparison between the predicted BOLD signal for a condition (namely, "Mirroring UD") using canonical HRF and FIR models at the MNI coordinate of an active voxel, overlaid on the measured BOLD time course. The gray dashed line represents the raw BOLD signal, while the blue and red lines show the model predictions using HRF and FIR basis sets, respectively. Notably, the advantage of multi-echo (ME) in terms of reliability, as observed with the canonical HRF, may be partly driven by a better fit to the assumed model rather than by more consistent underlying signal.

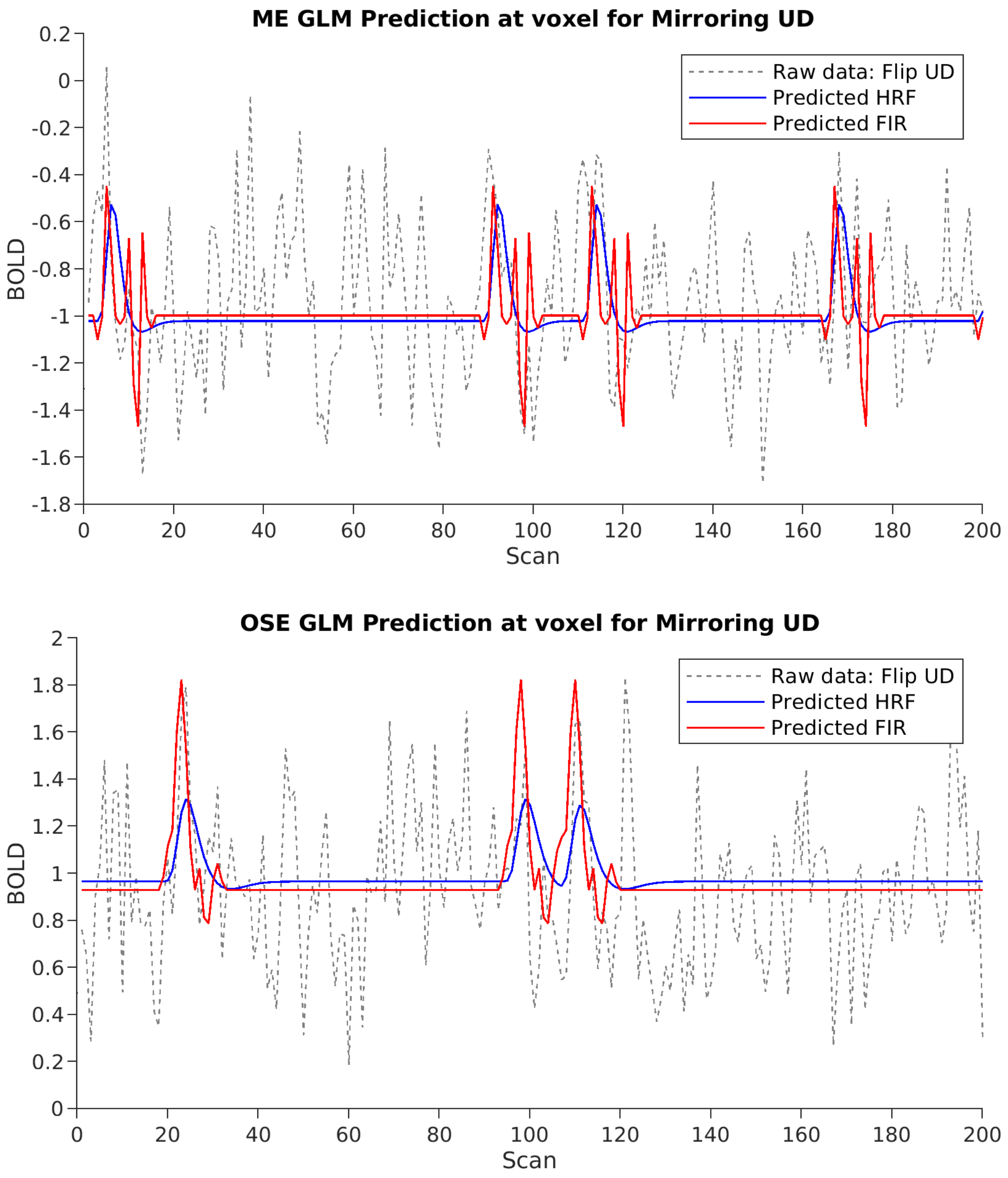

Figure S4. Beta weights and t-statistics comparison. Panel (a) shows the percent difference between single-echo data (either echo-2 or OSE) and ME, and panel (b) report the difference in t-statistics between single-echo data and ME.

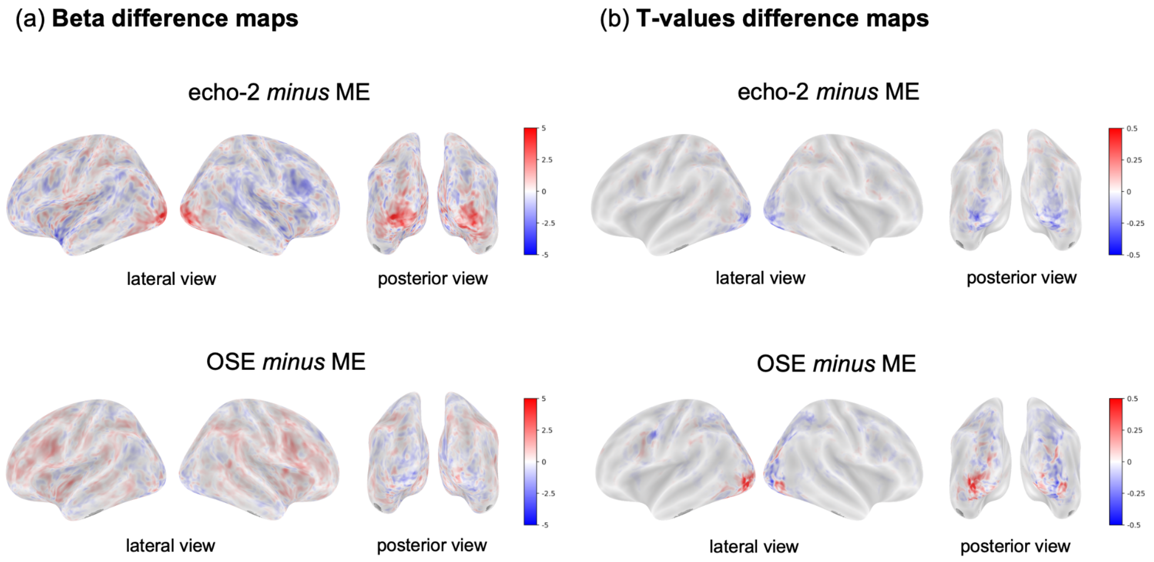

Table S1. Word stimuli presented during the reading task.
Stimuli consisted of 24 high-frequency three-syllable nouns selected from the Italian lexicon on the basis of (a) word structure (flat syllables or not; the presence of consonant clusters), (b) orthographic words, (c) length, and (d) category (abstract/concrete words).

Stimuli	Catch
lavoro	cavallo
governo	leone
amore	pesce
famiglia	gatto
mercato	
palazzo	
mattina	
cinema	
amico	
camera	
musica	
estate	
cultura	
bambino	
macchina	
natura	
ragazzo	
partita	
campagna	
capelli	

Table S2. Cluster extent threshold. Thresholds (in voxels) correspond to cluster-level FWE-corrected significance at $p < .05$, following a voxel-wise threshold of $p < .001$ uncorrected, as determined by SPM.

	**Cluster size threshold (FWE)**	
**fMRI contrast**	**ME-denoised**	**OSE**
**Mirroring LR**	44	19
**Mirroring UD**	21	20
**Mirroring LRUD**	27	23
**Rotation 45° & -45°**	45	17
**Rotation 15° & -15°**	-	54
**Rotation 30° & -30°**	21	29
**Spacing 2**	27	-
**Spacing 4**	26	18
**Spacing 6**	19	18
